# Web-Based Signal Detection Using Medical Forums Data in France: Comparative Analysis

**DOI:** 10.2196/10466

**Published:** 2018-11-20

**Authors:** Marie-Laure Kürzinger, Stéphane Schück, Nathalie Texier, Redhouane Abdellaoui, Carole Faviez, Julie Pouget, Ling Zhang, Stéphanie Tcherny-Lessenot, Stephen Lin, Juhaeri Juhaeri

**Affiliations:** 1 Epidemiology and Benefit Risk Evaluation Sanofi Chilly-Mazarin France; 2 Kappa Santé Paris France; 3 Kap Code Paris France; 4 Information Technology and Solutions Sanofi Lyon France; 5 Global Pharmacovigilance Sanofi Bridgewater, NJ United States; 6 Epidemiology and Benefit Risk Evaluation Sanofi Bridgewater, NJ United States

**Keywords:** adverse event, internet, medical forums, pharmacovigilance, signal detection, signals of disproportionate reporting, social media

## Abstract

**Background:**

While traditional signal detection methods in pharmacovigilance are based on spontaneous reports, the use of social media is emerging. The potential strength of Web-based data relies on their volume and real-time availability, allowing early detection of signals of disproportionate reporting (SDRs).

**Objective:**

This study aimed (1) to assess the consistency of SDRs detected from patients’ medical forums in France compared with those detected from the traditional reporting systems and (2) to assess the ability of SDRs in identifying earlier than the traditional reporting systems.

**Methods:**

Messages posted on patients’ forums between 2005 and 2015 were used. We retained 8 disproportionality definitions. Comparison of SDRs from the forums with SDRs detected in VigiBase was done by describing the sensitivity, specificity, positive predictive value (PPV), negative predictive value (NPV), accuracy, receiver operating characteristics curve, and the area under the curve (AUC). The time difference in months between the detection dates of SDRs from the forums and VigiBase was provided.

**Results:**

The comparison analysis showed that the sensitivity ranged from 29% to 50.6%, the specificity from 86.1% to 95.5%, the PPV from 51.2% to 75.4%, the NPV from 68.5% to 91.6%, and the accuracy from 68% to 87.7%. The AUC reached 0.85 when using the metric empirical Bayes geometric mean. Up to 38% (12/32) of the SDRs were detected earlier in the forums than that in VigiBase.

**Conclusions:**

The specificity, PPV, and NPV were high. The overall performance was good, showing that data from medical forums may be a valuable source for signal detection. In total, up to 38% (12/32) of the SDRs could have been detected earlier, thus, ensuring the increased safety of patients. Further enhancements are needed to investigate the reliability and validation of patients’ medical forums worldwide, the extension of this analysis to all possible drugs or at least to a wider selection of drugs, as well as to further assess performance against established signals.

## Introduction

Adverse drug reactions (ADRs) are an important public health concern. Drug safety currently depends on postmarketing surveillance, which is conducted through spontaneous reporting systems based on voluntary reports. The reporting rate is low, and the time for getting access to reported data can be long. Thus, it is difficult to detect signals of disproportionate reporting (SDRs) in a timely manner, and some may not even be captured by those reporting systems. Alternative sources of data have already been used to detect drug-adverse event (AE) associations, including claims data [1], electronic medical records (EMRs) [2-4], and consumer search logs [5,6]. In addition, approaches have been developed to combine SDRs from data sources such as EMRs, claims, internet search logs with SDRs from the Food and Drug Administration Adverse Event Reporting System (FAERS) [5,7,8].

With the development and popularity of social media and medical forums, many internet users are exchanging health-related information, which may involve ADRs, and the question of consistency and usefulness of these new data sources for Web-based signal detection is under scrutiny. Recent projects have aimed at investigating the quality of social media data, as well as investigating the most performant method for the Web-based signal detection. The use of Web-based data (such as query logs and social media) is emerging among regulators (Food and Drug Administration and European Medicines Agency), industry, and academia [5,6,8-10]. As an example, a public-private partnership between the European Commission and European Federation of Pharmaceutical Industries and Associations, called WEB-RADR: Recognising Adverse Drug Reactions has been launched in 2014; this consortium made of organizations, including European medicines regulators, academics, and the pharmaceutical industry, aims to develop new ways of gathering information on suspected ADRs. One of the objectives is to investigate the potential for publicly available social media data for identifying potential drug safety issues. A recently published study [11] has focused on the performance evaluation of established statistical signal detection algorithms in Twitter or Facebook for a broad range of drugs and adverse events. Another example is related to the recent collaboration between Sanofi and Microsoft [8]. In this study, a Web-based search query method, called a query log reaction score, was developed to detect whether AEs associated with certain drugs could be found from search engine query data. The results were compared with reference signal detection algorithms commonly used with the FAERS.

The potential strength of Web-based data relies on their volume and real-time availability, allowing early SDR detection. This study aims to assess the consistency of SDRs detected from patients’ forums in France and the ability to identify SDRs earlier than the traditional reporting systems. Three products were selected for this study (insulin glargine, teriflunomide, and zolpidem). SDRs detected from this Web-based source were compared with those detected from World Health Organization (WHO) AEs reporting system (VigiBase) using traditional SDR detection methods.

## Methods

### Data Sources

The sources of data used were (1) patients’ medical forums in France and (2) VigiBase, the WHO individual case safety reports (ICSR) database; these sources of data are described later.

### Medical Forum’s Messages Database: Detec’t

This was a retrospective study based on the secondary use of data from the Web—patients’ medical forums in France. The Detec’t database is a private database aggregating messages from social media, including safety information. We included 12 well-known medical forums in this study (see Multimedia Appendix 1). Every discussion publicly available at the time of the search and containing at least one message with the name of the drugs of interest (active substance or brand name) was extracted using a Web crawler (Detec’t Extractor). This messages scrapping was done by targeting patients' messages using the HTML structure of each forum. This Web crawler was then adapted for each data source. Messages were extracted with all metadata related to the message (date, author of the post ID, URL of the discussion, and name of the forum). Finally, data were cleaned (deletion of ads, quotation of other Web users, and signature) using the HTML structure of the posts.

Retrieved messages went through several steps of processing so that the drug-event pairs necessary to perform the analysis were obtained. First, messages were deidentified. The data processing then automatically ensured that the messages contained the name of the drugs of interest and the concept of drug use or intake to constitute the corpora of messages related to the drugs under study. These corpora were formatted and screened to detect all references to medical events referring to potential AE (ie, not the indication of the drug, not an AE preceding the intake of the drug, not a question about adverse effects without having experienced it, etc).

The deidentification of messages was performed using an in-house algorithm based on the regular expression to automatically identify specific sequences of characters (like proper names, phone numbers, postal codes, mail addresses, etc). Messages containing the names of the drugs were identified by automatically detecting, among the set of extracted messages, references to active substances, and brand names of the drugs of interest; this step included the detection of common spelling mistakes. Next, the drug intake notion was identified with a specific algorithm (on this set of messages) based on the detection of regular expressions (identifying first-person personal pronouns, for instance). This procedure ensured that the person experiencing the drug could be identified (this person could be the author of the post or one of their relative). These “intake messages” represented the set of messages on which the following steps were applied.

As described earlier [12], the formatting of messages included the conversion of messages to lowercases, the removal of extra whitespaces, and the tokenization of messages. All words from messages were stemmed using Porter’s algorithm to associate inflected and derived words together with their root form.

**Figure 1:**
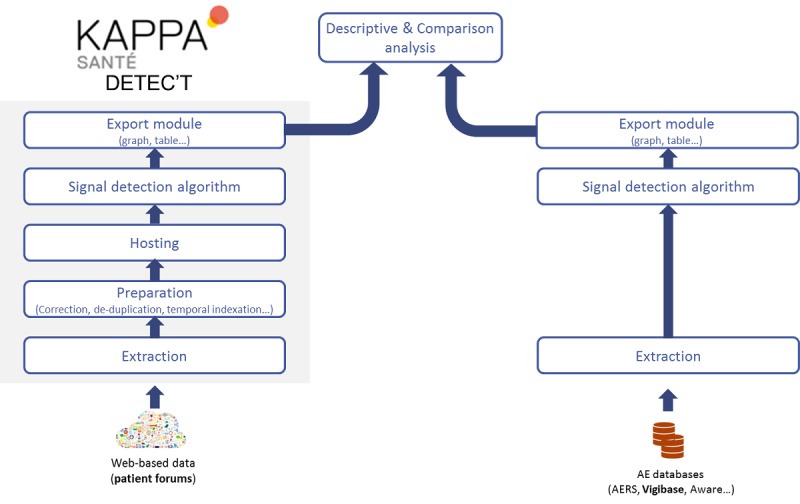
Extraction methodology and data preparation for analysis. AE: adverse events; AERS: Adverse Events Reporting System.

Medical concepts were detected using an extended version of Medical Dictionary for Regulatory Activities (MedDRA) version 15.0 adjusted and supplemented by vernacular vocabulary. In MedDRA, Preferred Terms (PT) correspond to unique descriptors of medical concepts. Lower-Level Terms (LLT) correspond to variants of PT and the lowest level of the terminology (each LLT is associated with a single parent PT and several LLTs can be associated with one PT). Medical concepts were detected at the LLT level and encoded as PT. The supplementation of vocabulary by vernacular terms was performed by manually reviewing a sample of messages from Web forums previously automatically annotated with MedDRA. The review was performed by 4 computer science experts who were familiar with the MedDRA dictionary. All medical concepts manually identified and not detected by MedDRA were saved and manually associated with a PT as if they were new LLTs. The evaluation of medical concepts detection, after supplementation on a sample of 157 messages, conducted to a recall of 71% and an accuracy of 93%. Regarding the adjustments provided to MedDRA, some PTs out of the study scope were removed (ie, “poverty” or “married”). For example, terms including the mention NOS (Not Otherwise Specified), that is, “Allergy NOS,” were cleaned by removing the mention. Eventually, all terms were stemmed. A manual cleaning was performed to deduplicate the terms obtained after stemming. The detection of medical concepts was performed by looking for exact matches between the stemmed versions of MedDRA and messages.

Potential AEs were identified by applying an algorithm [12] based on a Gaussian mixture model and then a support vector machine algorithm. This step identified drug-event pairs.

Extracted messages were stored in the database with (1) metadata associated with the messages; (2) results of the annotation process—drug intake, medical concepts, and their MedDRA code; and (3) the result of the AE detection algorithm.

The final step of the data preparation identified the number of messages for each drug-event pair (Figure 1).

### The Comparison Database: VigiBase

The comparison database used as the gold standard was VigiBase, the WHO Global ICSR database. It consists of reports of adverse reactions received from member countries since 1968. VigiBase is updated with incoming ICSRs on a continuous basis. The VigiBase data resource is the largest and most comprehensive in the world, and it is developed and maintained by the Uppsala Monitoring Centre on behalf of the WHO. By May 2015, over 11 million reports were available in the database. The VigiBase database system includes linked databases containing medical and drug classifications—WHO Adverse Reactions Terminology or MedDRA, WHO International Classification of Diseases, and WHO Drug Dictionary; these classifications enable structured data entry, retrieval, and analysis at different levels of precision and aggregation.

### Drugs

Three drugs (insulin glargine, teriflunomide, and zolpidem) were selected for the study to cover different therapeutic areas and different lengths of use since market authorization. Data corresponding to these selected drugs (active substance and brand name) were extracted from the Detec’t database. The following drugs were therefore searched on the database: insulin glargine AND glargine AND Lantus; teriflunomide AND Aubagio; zolpidem AND Stilnox. Synonyms of the drug name and spelling mistakes were considered (ie, detail on misspelling). Misspellings were added by identifying most common errors and adding them as researched forms of the drug.

In order to carry out the analysis of disproportionate reporting, a background group of 327 drugs was randomly selected from the Detec’t database. This later included drugs that were randomly chosen from an exhaustive list of French drugs. The messages corresponding to these 327 drugs were selected from the same set of forums and time period; they went through the same analysis and encoding steps.

### Signals of Disproportionate Reporting Detection Metrics and Definition

The disproportionality analysis of spontaneous reports (comparing the number of observed cases with that of expected cases) was used. The quantitative method in signal detection relies on the principle of disproportionality [7,13,14]. We used the Proportional Reporting Ratio (PRR), Reporting Odds Ratio (ROR), Reporting Fisher’s Exact Test (RFET), empirical Bayes geometric mean (EBGM), and the Information Component (IC). A total of 8 disproportionality definitions were considered for this study (Table 1).

### Statistical Analysis

#### Descriptive Analysis

A description of messages for the overall period (cumulative from 2005 to 2015) and across time within the study period was provided—numbers of messages with the drug name, numbers of messages containing the concept of drug use or intake, medical concepts, and potential AEs.

#### Comparative Analysis

The comparison of SDRs detected from patients’ forums in France to SDRs detected in VigiBase were described using sensitivity (true positive rate)=*a*/N1, specificity (true negative rate)=*d*/N2, positive predictive value (PPV)=*a*/M1, negative predictive value (NPV)=*d*/M2, and accuracy (*a*+*d*)/*N* (Tables 2 and 3). The receiver operating characteristics (ROC) curve and the area under the curve (AUC) were considered to measure the overall performance of the test to discriminate between positive and negative SDRs. The ROC curve represented the true positive rate (sensitivity) plotted in function of the false positive rate (100-specificity) for different thresholds of the metric.

**Table 1:** Definition of disproportionate signals.

Metric	Definition of disproportionate signal
Empirical Bayes Geometric Mean (EBGM)	EBGM≥2
Empirical Bayes Geometric Mean (EBGM)	EBGM≥4
Lower bound of the 95% CI of EBGM (EB05)	EB05≥2
Proportional Reporting Ratio (PRR)	PRR≥2, *N* ≥3, χ^2^≥4
Lower bound of the 95% CI of PRR (PRR025)	PRR025≥1
Lower bound of the 95% CI of the Reporting Odds Ratio (ROR025)	ROR025≥1
Lower bound of the 95% CI of the Information Component (IC025)	IC025=0
Reporting Fisher’s Exact Test (RFET) *P*	RFET *P*≤.05

**Table 2:** Signals: two-by-two contingency table for a combination of positive and negative signals from medical forums and VigiBase to measure performance.

Signals from medical forums	Signals from VigiBase		Total
	Positive	Negative	
Positive	a (true positive)	b (false positive)	M1
Negative	c (false negative)	d (true negative)	M2
Total	N1	N2	N

**Table 3:** Performance indicators.

Performance indicators	Value
Sensitivity (true positive rate)	*a*/N1
Specificity (true negative rate)	*d*/N2
Positive predictive value	*a*/M1
Negative predictive value	*d*/M2
Accuracy	(*a*+*d*)/*N*

#### Time Analysis

For SDRs identified in both data sources, we performed an analysis of time difference in months between the date of detection of SDRs from French patients’ forums and the date of detection of SDRs from VigiBase.

## Results

### Descriptive Analysis

**Figure 2:**
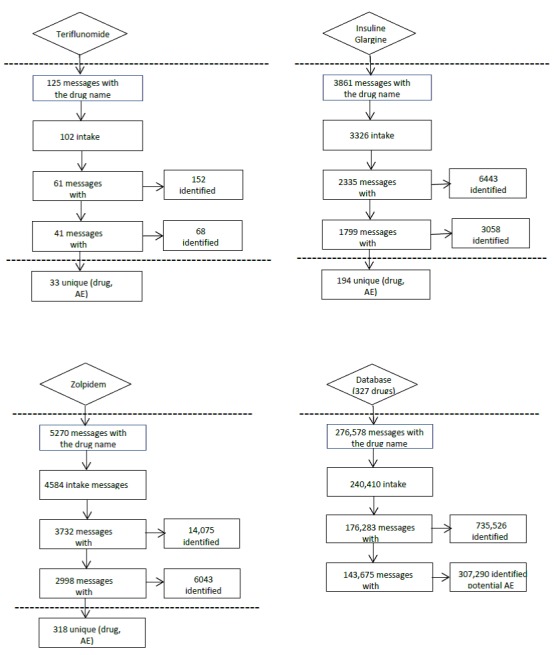
Flowchart for the 3 drugs and the other 327 drugs (comparison group). AE: adverse events.

The data from 8 medical forums were considered for analysis and corresponded to messages published between January 1, 2005 and December 31, 2015, for insulin glargine and zolpidem and between April 1, 2014 and December 31 2015, for teriflunomide. For teriflunomide, this time restriction was because the product received its first marketing authorization application in September 2013 and was first launched in March 2014. Figure 2 shows the messages flowchart. The extraction was conducted for the identification of 102 messages for teriflunomide, 3326 messages for insulin glargine, and 4584 messages for zolpidem, which were relative to the drug intake. Among those, 61 messages for teriflunomide, 2335 messages for insulin glargine, and 3732 messages for zolpidem contained medical concepts. Among those, 41 messages for teriflunomide, 1799 messages for insulin glargine, and 2998 messages for zolpidem contained potential AEs. This resulted in 33 unique drug-event pairs for teriflunomide, 194 for insulin glargine, and 318 for zolpidem. The number of SDRs detected varied across the definition of disproportionate SDRs. For teriflunomide, insulin glargine and zolpidem, the number of SDRs varied from 4 to 12, 21 to 48, and 23 to 95, respectively. These SDRs were compared with the SDRs detected in VigiBase.

### Comparative Analysis

**Figure 3:**
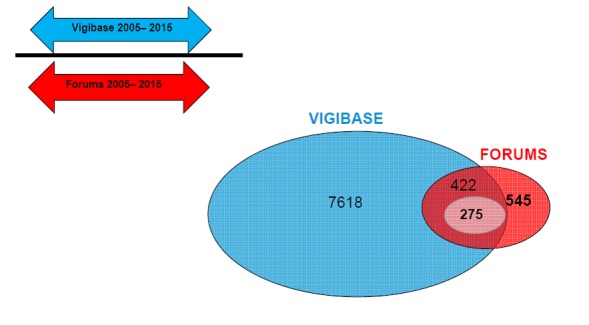
Time periods covered by VigiBase and the forums database and the number of drug-event pairs overlap, as well as pairs overlap with at least 2 messages (smallest circle).

Between 2005 and 2015, the medical forums data contained 545 drug-event pairs. Overall, 7618 pairs were identified in VigiBase, of which 422 drug-event pairs combinations overlapped with the forums data (Figure 3). The overlap considered an exact match with the event terminology. When restricting to pairs with at least 2 messages, the overlap was 275 drug-event pairs.

Among 545 drug-event pairs from the forums, only 123 were not identified in VigiBase; those 123 drug-event pairs corresponded each to only one message in the forums. The individual inspection showed that some PTs were not adequately specific. For example, some PTs identified in the forums were “weight,” but it was not possible to match them with the PT, such as “underweight” or “overweight” or “abnormal weight gain” or “weight abnormal,” which were identified in the drug-events pairs from VigiBase.

Among the overlap of 422 drug-event pairs, the specificity was high (87.5%-95.5%) depending on the SDR definition (Table 4). On the other hand, the sensitivity was low (29%-50.6%), indicating that an important proportion of SDRs from VigiBase was not identified in the forums. The PPV (51.2%-75.4%), NPV (68.5%-91.6%), and accuracy (68%-87.7%) were high.

Among 275 drug-event pairs (for which at least 2 reports or messages were considered; Table 5), the figures were slightly higher, improving the overall performance. The specificity varied between 86.9% and 93.4% and the sensitivity between 39.1% and 56.5%. The PPV (53.9%-76.6%), NPV (68.7%-91.5%), and accuracy (70.6%-86.2%) were high.

**Figure 4:**
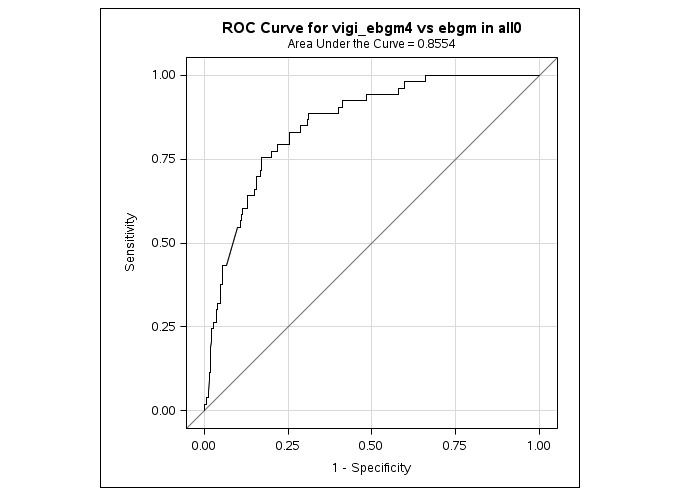
The receiver operating characteristics (ROC) curves and area under the curve applying empirical Bayes geometric mean (EBGM)≥4 in the VigiBase and EBGM in the forums.

Whatever definition of disproportionate SDR used, the ROC curves and the AUC showed an overall good performance. AUC varied around 0.8. The highest AUC was shown with the EBGM metric (AUC=0.85; Figure 4).

### Time Analysis

For SDRs detected both in VigiBase and patients’ forums, we calculated the time difference in months between the date of detection of positive SDRs from the French patients’ forums and the date of detection of these SDRs in VigiBase (Table 6).

**Table 4:** The sensitivity, specificity, positive predictive value, negative predictive value, and accuracy among 422 drug-event pairs.

Definition	Sensitivity (%)	Specificity (%)	Positive predictive value (%)	Negative predictive value (%)	Accuracy (%)
EB05^a^≥2	29.0	95.5	62.5	84.0	82.0
EBGM^b^≥2	48.2	89.3	62.5	82.3	78.2
EBGM≥4	39.6	94.6	51.2	91.6	87.7
PRR^c^≥2, *N*≥3, χ^2^≥4	31.9	94.0	67.9	77.9	76.5
Lower 95% CI of PRR≥1	37.3	87.5	64.1	70.0	68.7
Lower 95% CI of ROR≥1	37.0	87.9	66.3	68.5	68.0
IC025^d^>0	33.3	94.2	75.4	72.5	73.0
RFET^e^: *P*≤.05	50.6	86.1	68.1	74.8	73.0

^a^EB05: Lower bound of the 90% CI of empirical Bayes geometric mean.

^b^EBGM: empirical Bayes geometric mean.

^c^PRR: Proportional Reporting Ratio.

^d^IC025: Lower bound of the 95% CI of the information component.

^e^RFET: Reporting Fisher’s Exact Test.

**Table 5:** The sensitivity, specificity, positive predictive value, negative predictive value, and accuracy among 275 drug-event pairs.

Definition	Sensitivity (%)	Specificity (%)	Positive predictive value (%)	Negative predictive value (%)	Accuracy (%)
EB05^a^≥2	39.1	93.4	64.1	83.5	80.7
EBGM^b^≥2	49.4	88.5	65.1	80.2	76.7
EBGM≥4	51.2	92.3	53.9	91.5	86.2
PRR^c^≥2, *N*≥3, χ^2^≥4	44.2	91.5	70.4	78.3	76.7
Lower 95% CI of PRR≥1	48.3	88.7	75.7	70.2	71.6
Lower 95% CI of ROR≥1	47.1	88.5	75.7	68.7	70.6
IC025^d^>0	45.8	91.1	76.6	72.5	73.5
RFET^e^: *P*≤.05	56.5	86.9	75.6	73.6	74.2

^a^EB05: Lower bound of the 90% CI of empirical Bayes geometric mean.

^b^EBGM: empirical Bayes geometric mean.

^c^PRR: Proportional Reporting Ratio.

^d^IC025: Lower bound of the 95% CI of the information component.

^e^RFET: Reporting Fisher’s Exact Test.

**Table 6:** The time difference in months of signals detection dates (∆time) between patients’ forums and VigiBase.

Definition	∆time^a^<0, n (%)	∆time^a^=0, n (%)	∆time^a^>0, n (%)	Total number of pairs, n (%)
PRR^b^≥2, *N*≥3, χ^2^≥4	15 (25.4)	3 (5.1)	41 (69.5)	59 (100)
EB05^c^≥2	10 (32.3)	3 (9.7)	18 (58.1)	31 (100)
EBGM^d^≥2	22 (26.5)	4 (4.8)	57 (68.7)	83 (100)
EBGM≥4	12 (37.5)	4 (12.5)	16 (50)	32 (100)
IC025^e^>0	13 (21.3)	3 (4.9)	45 (73.8)	61 (100)
Lower 95% CI of PRR≥1	29 (32.6)	5 (5.6)	55 (61.8)	89 (100)
Lower 95% CI of ROR≥1	29 (32.2)	5 (5.6)	56 (62.2)	90 (100)
RFET^f^: *P*≤.05	34 (30.6)	7 (6.3)	70 (63.1)	111 (100)

^a^∆time: detection date in patients’ forums−detection date in VigiBase.

^b^PRR: Proportional Reporting Ratio

^c^EB05: Lower bound of the 90% CI of empirical Bayes geometric mean.

^d^EBGM: empirical Bayes geometric mean.

^e^IC025: Lower bound of the 95% CI of the information component.

^f^RFET, Reporting Fisher’s Exact Test.

Depending on the definition of SDRs, up to 38% (12/32) of common SDRs were detected earlier (up to 128 months earlier) in the forums than in VigiBase. In addition, up to 13% (4/32) were detected at the same date but were available earlier in the forums given the real-time availability of data on the Web. The qualitative exploration of SDRs detected earlier in the forums showed heterogeneity as some were related to serious medical events and other to patients-related symptoms (ie, stress and hunger).

Most signals that were detected earlier in VigiBase were linked to serious medical events, which probably led to medical consultation and, thus, to an AE reporting done through a health care professional. In addition, most of those events were related to the System Organ Class “nervous system disorders” and “psychiatric disorders.”

## Discussion

### Principal Findings

This study aimed at assessing the consistency of SDRs detected from patients’ forums in France over the last 11 years and the ability to identify SDRs earlier than that in VigiBase.

The potential strength of Web-based data relies on their volume and real-time availability, allowing early signal detection. This pilot study showed a good performance and earlier detection of SDRs in the French medical forums compared with SDRs detected in traditional sources. In addition, these pilot results indicate that using patients’ medical forums may be considered as a complementary source of data to traditional sources, allowing SDRs to be detected earlier and, thus, facilitating the increased safety of patients.

We first compared SDRs (by considering several definitions of disproportionate SDRs) detected in the forums data and the WHO AEs reporting system (VigiBase). The comparison of positive and negative SDRs showed that whatever the definition of disproportionate SDR, the sensitivity was low and the specificity was very high. In addition, the PPV and NPV were high. The overall performance was good, showing that data from the medical forum may be a valuable source to be considered for signal detection. In another study [8] using query log data, results showed that the method had moderate sensitivity and low specificity in detecting signals in Web query data compared with reference signal detection algorithms in FAERS. In another study [11] using Twitter and Facebook, the authors suggested that broad-ranging statistical signal detection in Twitter and Facebook, using currently available methods for adverse event recognition, performs poorly and cannot be recommended at the expense of other pharmacovigilance activities; this indicates that results in terms of performance might vary according to the Web data source used and the metric used for SDRs.

Second, among SDRs from patients’ forums and VigiBase, we calculated time differences in detection of SDRs to measure the ability of forums data to detect earlier SDRs compared with VigiBase. Up to 38% (12/32) of common SDRs could be detected earlier when using the forums data, which is an important finding. The qualitative exploratory analysis of the SDRs detected earlier showed that events were related to serious as well as patient-related symptoms. This finding is consistent with recent studies [15] that addressed the question of earlier detection of drug-related AEs in the social media compared with FAERS. The findings highlighted some of the promises of social media data sources for detecting early AE reports patterns compared with conventional pharmacovigilance sources and showed that social media AE reports helped predict the occurrence of FAERS reports several months later for one of the two drugs that were studied. In a study [16], the objective was to examine whether specific product-AE pairs were reported through social media before being reported to FAERS. In one of the positive cases, the first report occurred in social media prior to the SDR detection from FAERS. Authors concluded that an efficient semiautomated approach to social media monitoring might provide earlier insights into certain AEs.

### Strengths and Limitations

One of the strengths of this study was the quality of preprocessing and processing of the data extracted from the forums. Messages that were used for Web-signal detection in this study were not only containing the drug but also a medical event (cooccurrence) as this is done in other studies [17]. Several cleansing and validation steps were performed to ensure that the identified messages were related to an internet user who used the concept of use or intake of the drug and with potential AE.

This study has several limitations. First, the results only apply to 3 drugs and for the French medical forums. Thus, results are not generalizable to all drugs and at a worldwide scale. However, we do not have a strong hypothesis to believe that the use of Web-based medical forums and interactions of French internet users would be different in other developed countries. Thus, further studies focusing on worldwide patients’ forums should be envisaged. Second, automatics algorithms have their limitations. The management of Web-based data needs continuous updates on modeling and data processing to ensure high quality and accuracy of the information retrieved. Although the data were processed, it is still possible that some drug names or medical concepts could be missed, that some AEs may be confused with drugs indications, or questions from Web users about AEs they have not experienced, or descriptions of symptoms that are not adverse reactions to drugs. Third, Web-based data rely on patients’ perspective and declaration but not on a true medical diagnosis. Web-based data are sensitive to increase in the media coverage, resulting in increased searches or posts and are prone to changes in people’s search or communication behavior. Finally, VigiBase is not a true gold standard, as it has its own limitations (such as lack of denominator and underreporting). VigiBase, however, has been used as a standard for signal detection by regulators and pharmaceutical companies, and our study showed that patients’ forum could be used as a complementary data source to detect SDR earlier. Although the choice of the reference data remains challenging [18], further studies using a refined gold standard, such as drug-event pairs shown in the labels, should be considered.

### Conclusions

This study shows a good performance and earlier detection of SDRs detected in patients’ medical forums compared with SDRs detected in traditional sources. Those SDRs relate to serious medical events as well as subjective patients-related symptoms (eg, stress and hunger). These results indicate that using patients’ medical forums may be considered as a complementary source of data to traditional sources, allowing SDRs to be detected earlier and, thus, ensuring the increased safety of patients. Further enhancements are needed to investigate the reliability and validation of patients’ medical forums worldwide, the extension of this analysis to all possible drugs or at least to a wider selection of drugs, as well as to further assess performance against established signals.

## Appendix

Multimedia Appendix 1 List of medical forums included in the study.

## Abbreviations

AE: adverse event
AERS: Adverse Events Reporting System
AUC: area under the curve
EBGM: empirical Bayes geometric mean
FAERS: Food and Drug Administration Adverse Event Reporting System
IC: information component
ICSR: individual case safety reports
LLT: lower-level terms
NOS: Not Otherwise Specified
NPV: negative predictive value
PPV: positive predictive value
PRR: proportional reporting ratio
PT: preferred terms
RFET: Reporting Fisher’s Exact Test
ROC: receiver operating characteristics
ROR: reporting odds ratio
SDR: signals of disproportionate reporting
WHO: World Health Organization
MedDRA: Medical Dictionary for Regulatory Activities
