# Supplementary material for: Web-Based Signal Detection Using Medical Forums Data in France: Comparative Analysis
Source: J Med Internet Res. 2018 Nov 20;20(11):e10466. doi: 10.2196/10466 (PMC6280030; doi:10.2196/10466)
Supplement: Multimedia Appendix 1 [file jmir_v20i11e10466_app1.pdf]

Multimedia Appendix 1. List of medical forums included in the study.

Atoute	www.atoute.org/n/forum
Doctissimo	http://forum.doctissimo.fr/
E-sante	www.forum.e-sante.fr
SanteMedecine	http://sante-medecine.commentcamarche.net/forum
Onmeda	http://www.onmeda.fr/forum/
Futura sciences	http://forums.futura-sciences.com/
Psoriasis	www.forum-psoriasis.medicalistes.org/
AlarmAsso	http://alarme.asso.fr/forum/
Morphee	http://morphee.forumpro.fr/
Albi	http://forum.albifrance.org/
Renaloo	http://www.renaloo.com/forum/
Allodocteurs	http://www.allodocteurs.fr/forums-et-chats/forums
